# Giant Dielectric Properties of W^6+^-Doped TiO_2_ Ceramics

**DOI:** 10.3390/molecules27196529

**Published:** 2022-10-02

**Authors:** Porntip Siriya, Pairot Moontragoon, Pornjuk Srepusharawoot, Prasit Thongbai

**Affiliations:** 1 Giant Dielectric and Computational Design Research Group (GD–CDR), Department of Physics, Faculty of Science, Khon Kaen University, Khon Kaen 40002, Thailand; 2 Institute of Nanomaterials Research and Innovation for Energy (IN–RIE), Khon Kaen University, Khon Kaen 40002, Thailand

**Keywords:** giant dielectric response, TiO_2_, W^6+^, sintering, impedance spectroscopy

## Abstract

The effects of the sintering temperature and doping level concentration on the microstructures, dielectric response, and electrical properties of W^6+^-doped TiO_2_ (WTO) prepared via a solid-state reaction method were investigated. A highly dense microstructure, pure rutile-TiO_2_, and homogenously dispersed dopant elements were observed in all of the ceramic samples. The mean grain size increased as the doping concentration and sintering temperature increased. The presence of oxygen vacancies was studied. A giant dielectric permittivity (ε′ ~ 4 × 10^4^) and low tanδ (~0.04) were obtained in the WTO ceramic sintered at 1500 °C for 5 h. The ε′ response at a low temperature was improved by increasing the doping level concentration. The giant ε′ response in WTO ceramics can be described by the interfacial polarization at the interface between the semiconducting and insulating parts, which was supported by the impedance spectroscopy.

## 1. Introduction

In the field of electronic materials, dielectric materials have potential applications in electronic devices such as capacitors and memory devices. A large number of dielectric materials with large dielectric permittivities (ε′ > 10^4^) have been studied in recent years. Many giant ε′ materials, such as CaCu_3_Ti_4_O_12_ (CCTO) and related compounds [1,2,3,4,5], NiO_2_-based oxides [6], and Ln_2-x_Sr_x_NiO_2_ (where Ln = Nd, La) [7] have been widely studied. Nevertheless, the loss tangent (tanδ) of these materials is high and they are not appropriately used in capacitor applications.

Recently, high-performance dielectric properties, i.e., ε′ > 10^4^, tanδ < 0.05, and temperature stability of ε′ in the temperature range of 80–450 K, were reported in In^3+^/Nb^5+^ co-doped TiO_2_ ceramics [8]. The origin of the giant ε′ response and very low tanδ was proposed by a new mechanism, i.e., the electron-pinned defect-dipole (EPDD) effect. However, the internal barrier layer capacitor (IBLC) [9,10,11] and surface barrier layer capacitor (SBLC) effects were suggested to be the origin of the giant ε′ [12]. In addition, good dielectric properties have been observed in many acceptor/donor (A/D) co-doped TiO_2_ systems, such as Al^3+^/Nb^5+^ [13,14,15], Al^3+^/Ta^3+^ [16], Ga^3+^/Nb^5+^ [17], Eu^3+^/Nb^5+^ [18], Eu^3+^/Ta^5+^ [19], Nd^3+^/Nb^5+^ [20], Nd^3+^/Ta^5+^ [21], La^3+^/Nb^5+^ [22], Gd^3+^/Nb^5+^ [23], Bi^3+^/Sb^5+^ [24], Mg^2+^/Nb^5+^ [25], Ag^+^/Nb^5+^ [26], and Ag^+^/Ta^5+^ [27] co-doped TiO_2_ ceramics.

One of the most interesting giant dielectrics of TiO_2_-based ceramics is A/W^6+^ co-doped TiO_2_ ceramics [28,29,30]. For example, Ag^+^/W^6+^ co-doped TiO_2_ (AWTO) ceramics exhibited a high ε′ > 10^4^ and low tanδ (<0.05). Furthermore, the temperature coefficient was <±15% in the temperature range from −80 to 200 °C. The large ε′ response in the AWTO ceramics can be attributed to the IBLC effect. Up to now, there are fewer reports on W^6+^-doped TiO_2_ than A/D^5+^.

Most recently, Tuichai et al. studied the dielectric properties of 1%(Cr^3+^/Ta^5+^) co-doped TiO_2_ sintered at various temperatures [31]. The average grain size and ε′ value significantly increased with an increase in the sintering temperature, while tanδ decreased. The large ε′ response can be described by the space charge polarization, while the low tanδ was caused by the high resistance of the insulating layers with very large potential barrier height. Moreover, in previous studies [8,17], the excellent dielectric properties were improved by optimizing the sintering condition coupled with the doping concentration. To the best of our knowledge, a giant dielectric response with a low tanδ in single-doped TiO_2_ has rarely been reported. The aim of this study is to investigate a new single-doped TiO_2_ that can exhibit a large ε′ value and low tanδ.

In this study, the dielectric and electrical properties of W^6+^-doped TiO_2_ with various W^6+^ doping levels and sintering temperatures were studied. The crystal structure, phase composition, and microstructure were analyzed. Notably, a giant ε′ of ~4 × 10^4^ and low tanδ (~0.04) were achieved. The giant ε′ response is discussed.

## 2. Experimental Details

Single-doped W_x_Ti_1-x_O_2_ ceramics with x = 0.0025 (0.25%WTO), 0.005 (0.5%WTO) and 0.25 (2.5%WTO) were prepared by the wet-balling method. TiO_2_ (Sigma-Aldrich, >99.9% purity) and WO_3_ (Fluka, 99.9% purity) were used as starting raw materials. Details of the preparation route have been provided in previous studies [32]. The mixed powders were formed into pellets. The 0.25%WTO sample was obtained by sintering at 1400 °C for 5 h, while the 2.5%WTO sample was sintered at 1200, 1300, 1400, and 1500 °C for 5 h. The 2.5%WTO ceramics sintered at these temperatures were referred to as the 2.5%WTO-1200, 2.5%WTO-1300, 2.5%WTO-1400, and 2.5%WTO-1500 ceramics, respectively. The 0.25%WTO and 0.5%WTO sintered at 1500 °C for 5 h were referred to as the 0.25%WTO-1500 and 0.5%WTO-1500 ceramics, respectively.

The sintered samples were characterized using field emission scanning electron microscopy (FESEM, Helios NanoLab, G3 CX), energy-dispersive X-ray analysis (EDS-mapping), X-ray diffraction (XRD, PANalytical, EMPYREAN), and Raman spectroscopy (Bruker, Senterra II). The surfaces of the as-sintered ceramics were carefully cleaned before characterization of the surface using the SEM and EDS techniques.

The electrodes of all ceramic samples (not polished) were coated with Au through sputtering at a current of 25 mA for 8 min using a Polaron SC500 sputter-coating unit (Sussex, UK). The dielectric response of the ceramic samples was measured under an AC oscillation voltage of 0.5 V using an impedance analyzer (KEYSIGHT, E4990A) over a frequency range of 10^2^–10^6^ Hz. The temperature dependence of the dielectric properties was measured in the temperature range −60 to 210 °C. The impedance calculations of all the ceramic samples were as follows:

(1) $$Z^{*}=\;Z\prime -{\mathrm{jZ}}^{\prime \prime }=\frac{1}{R_{g}^{-1}+{\;j\omega C}_{g}}+\frac{1}{R_{\mathrm{gb}}^{-1}+{j\omega C}_{\mathrm{gb}}}$$

where C_gb_ and C_g_ are the capacitances of the grain boundaries and grain, respectively. R_gb_ and R_g_ are the resistance of the grain boundaries and grain, respectively.

## 3. Results and Discussion

**Figure 1:**
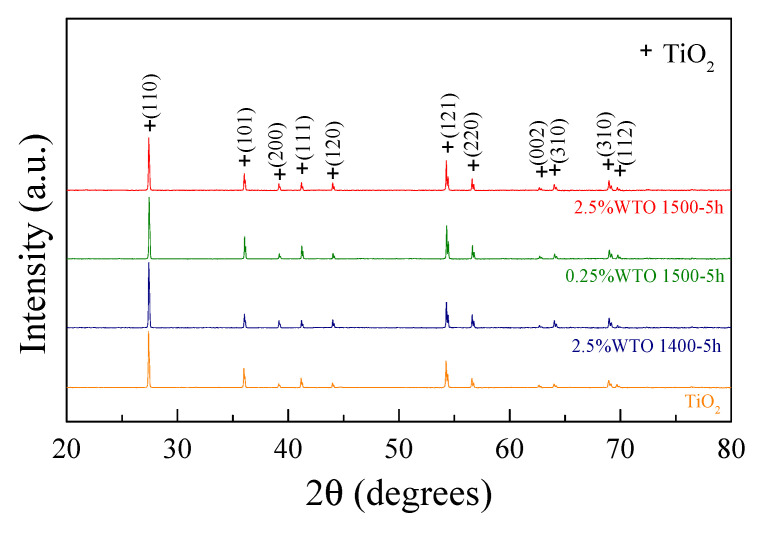
XRD patterns of TiO_2_ and WTO ceramics.

Figure 1 shows the XRD patterns of the WTO ceramic samples. The rutile-TiO_2_ phase (JCPDS 21-1276), based on a tetragonal structure with no impurity phase, was obtained. Lattice parameters (*a* and *c*) were calculated from the XRD patterns. The *a* and *c* values are summarized in Table 1. The ionic radii of W^6+^ (r_6_ = 60.0 pm) and Ti^4+^ (r_6_ = 60.5 pm) [33] are slightly different. Thus, the *a* and *c* values of the WTO ceramics are slightly changed when compared to those of pure TiO_2_. Moreover, the lattice parameters slightly changed as the co-doping concentration increased. These results (e.g., slightly changed lattice parameters and the absence of a secondary phase) indicate that the W^6+^ can enter into the Ti site owing to slightly different ionic sizes between them. The *a* and *c* values slightly change with the doping and sintering conditions. This result is similar to that reported in a previous study [31].

**Figure 2:**
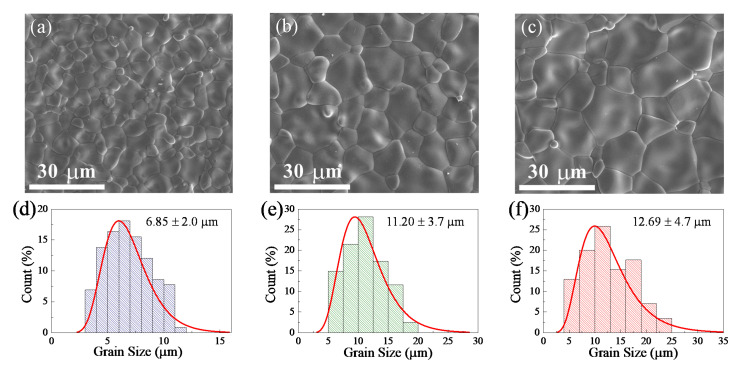
SEM images of the (**a**) 2.5%WTO-1400, (**b**) 0.25%WTO-1500, and (**c**) 2.5%WTO-1500 ceramics. Grain size distribution of the (**d**) 2.5%WTO-1400, (**e**) 0.25%WTO-1500, and (**f**) 2.5%WTO-1500 ceramics.

**Figure 3:**
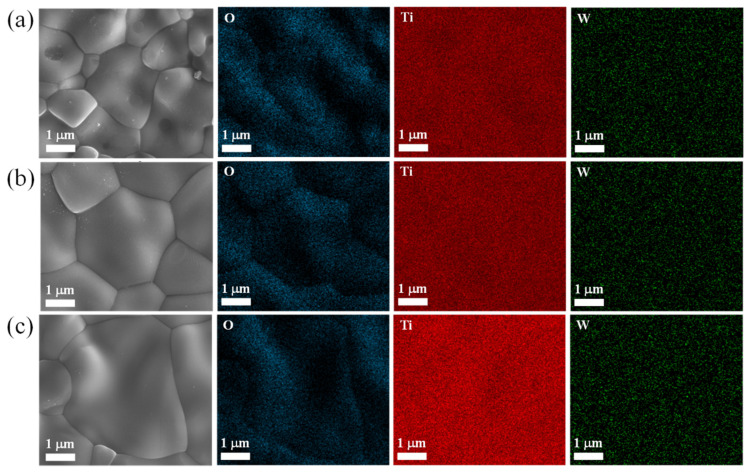
SEM-EDS mapping of the (**a**) 2.5%WTO-1400, (**b**) 0.25%WTO-1500, and (**c**) 2.5%WTO-1500 ceramics.

The morphologies and grain size distributions are illustrated in Figure 2a–f, respectively. Highly dense microstructures were obtained. The average grain sizes of the 2.5%WTO-1400, 0.25%WTO-1500, and 2.5%WTO-1500 ceramics were approximately 6.85 ± 1.98 μm, 11.20 ± 3.66 μm, and 12.69 ± 4.71 μm, respectively. The average grain size increased slightly when the doping concentration was increased. Nevertheless, the mean grain size of the sample increased significantly when the sintering temperature was increased. This effect of the sintering conditions on the grain size was similarly reported in the previous literature [31]. According to the EDS results, the dopant (i.e., W) was well-dispersed in the grain and grain boundaries, as shown in Figure 3. Accordingly, the wt.% ratios of W/Ta for the 2.5%WTO-1400, 0.25%WTO-1500, and 2.5%WTO-1500 ceramics were 1.9, 1.7, and 2.3%, respectively. The variations in the ratio may be due to the inhomogeneity of the sintered samples.

**Figure 4:**
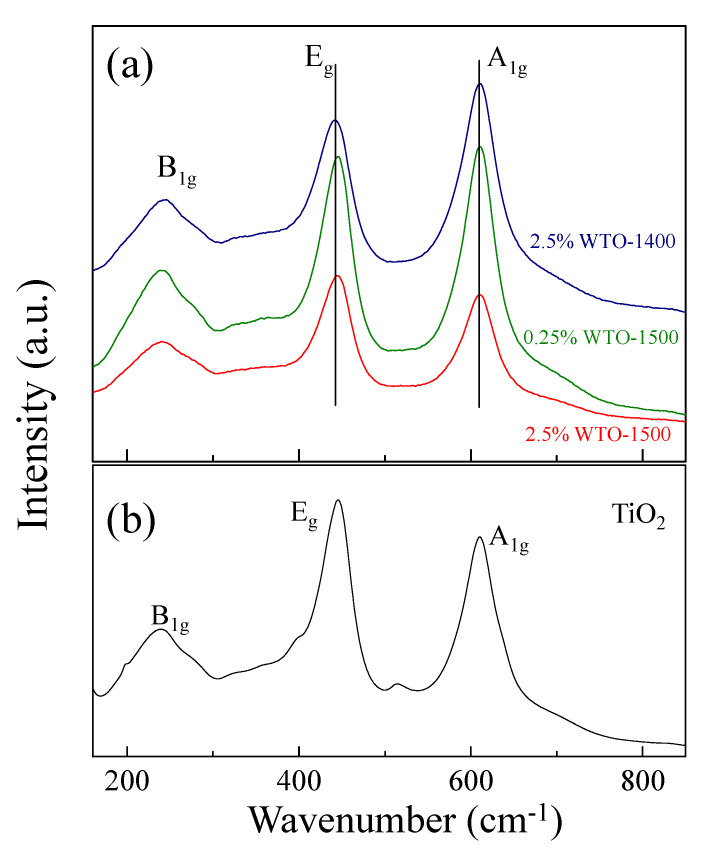
Raman spectra of (**a**) WTO ceramics and (**b**) pure TiO_2_ceramic.

According to the SEM results, the grain growth of WTO ceramics is likely influenced by the diffusion of oxygen ions during sintering. Thus, to confirm the presence of oxygen vacancies in the structure, the shifting of the Raman peak of WTO ceramics was compared with that of a pure-TiO_2_ ceramic. Figure 4a displays the Raman spectra of the WTO ceramics. The Raman peaks of the B_1g_, A_1g_, and E_g_ modes refer to O-Ti-O bond bending, Ti-O stretch modes, and oxygen vacancies, respectively [13,15,16,28,34]. In this study, we focused on the changes in the A_1g_ and E_g_ peaks. The wavenumbers of ~610.5 cm^−1^, 610.5 cm^−1^, and 611.0 cm^−1^ refer to the A_1g_ modes of the 2.5%WTO-1400, 0.25%WTO-1500, and 2.5%WTO-1500 ceramics, respectively. The E_g_ modes of 2.5%WTO-1400, 0.25%WTO-1500, and 2.5%WTO-1500 ceramics appeared at the wavenumbers of ~442.0 cm^−1^, 445.5 cm^−1^, and 444.5 cm^−1^, respectively. As shown in Figure 4b, compared with the pure TiO_2_ (A_1g_ ~ 610.0 cm^−1^ and E_g_ ~ 447.0 cm^−1^), the peak of A_1g_ mode is slightly shifted to a higher wavenumber due to the changing of the O-Ti-O bounds. This result is consistent with the XRD result because replacing Ti^4+^ with W^6+^ caused the O-Ti-O bonds and the lattice parameters to be slightly changed. For the E_g_ mode, the peaks of the E_g_ mode are shifted to a lower wavenumber compared to the pure rutile-TiO_2_ ceramic. This result indicates that the oxygen vacancies detected in the WTO structure were caused by oxygen loss during the sintering [11], which can be expressed as follows:

(2) $$O_{O}\;\to \;\frac{1}{2}O_{2}+\;V_{O}^{••}+2e^{\prime }$$

Besides the sintering effect at a high temperature, it is possible that W^3+^ might be formed, but this has not been proved. If so, $V_{O}^{••}$ can also be produced by the W^3+^ doping ions. Usually, oxygen vacancies in co-doped TiO_2_ systems can be produced by acceptor doping (e.g., In, Ga, Sc, and Mg) [8,30,35]. Thus, as this work has no acceptor dopant, the oxygen vacancies in WTO ceramics can only be due to the sintering process. Thus, the significantly increased average grain size of the 2.5%WTO-1500 ceramic, compared to that of the 2.5%WTO-1400 ceramic, should be attributed to the enhanced diffusion coefficient of $V_{O}^{••}$ when the sintering temperature increased. This gave rise to the increased grain boundary mobility, leading to an enlarged grain size.

**Figure 5:**
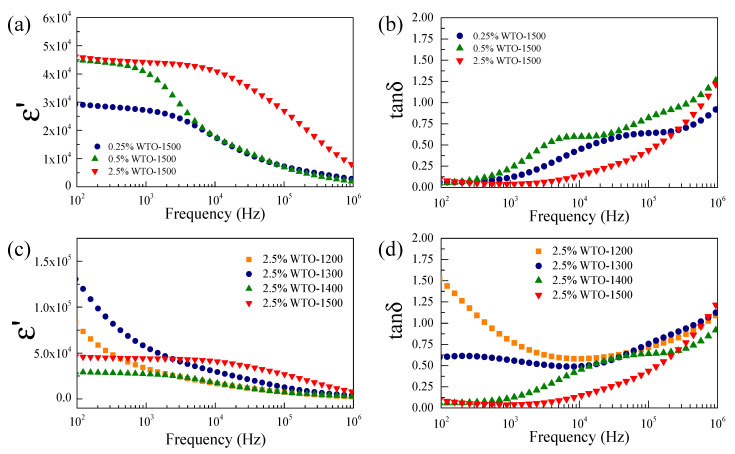
(**a**,**b**) Frequency dependence of ε′ and tanδ at room temperature for WTO ceramics doping with W^6+^ concentration (sintered at 1500 °C for 5 h). (**c**,**d**) Frequency dependence of ε′ and tanδ at room temperature for 2.5%WTO sintered at different temperatures (1200–1500 °C).

Figure 5a shows the effect of doping concentrations on the dielectric properties (at 30 °C, 10^2^–10^6^ Hz) of the WTO ceramics sintered at 1500 °C for 5 h. The ε′ of the 2.5%WTO-1500 ceramic reached a frequency of 10^4^ Hz, whereas the ε′ of the 0.25%WTO-1500 and 0.5%WTO-1500 ceramics rapidly decreased when the frequency increased higher than 10^3^ Hz. The decrease in ε′ correlates with the relaxation peak of tanδ, as shown in Figure 5b. The plateau of a giant ε′ extended to a high-frequency range as the doping concentration increased. This result indicates that the giant dielectric properties of the WTO ceramics can be improved by the optimization the doping concentrations. The defect concentration usually increases with the increase in the doping concentration, giving rise to an increased frequency range of the giant ε′ response. For the 0.25%WTO-1500 and 0.5%WTO-1500 ceramics, a step-like decrease in ε′ (f > 10^3^) correlated with the relaxation peak of tanδ. This ε′ response may have originated primarily from the IBLC effect. Moreover, ε′ ~ 10^3^ in the high-frequency range arises from the intrinsic value of rutile-TiO_2_ [20,36,37].

Figure 5c,d show the dielectric properties of the 2.5%WTO ceramics sintered at various temperatures from 1200 to 1500 °C. The ε′ of the 2.5%WTO-1200 and 2.5%WTO-1300 was strongly dependent on the frequency over the measured range, which was accompanied by large values of tanδ. These results are attributed to the effect of the sample–electrode interface related to the long-range motion of free charges. Notably, the plateau of a giant ε′ of the 2.5%WTO ceramics was obtained using the sintering temperature of >1400 °C for 5 h. The ε′ value further increased with the increase in the sintering temperature from 1400 to 1500 °C. Furthermore, the plateau of a giant ε′ extended to a high-frequency range as the sintering temperature increased. A low tanδ of the 2.5%WTO-1500 ceramic was obtained in a wide frequency range compared to those of other samples. For the 2.5%WTO-1400 ceramic, two step-like decreases in ε′ were observed at low temperatures (<10^3^ Hz) and high temperatures (>10^5^ Hz), which are related to the tanδ peak. The step-like decrease in ε′ that appeared at a low temperature (<10^3^ Hz) was caused by the SBLC effect, while the giant ε′ response in the frequency range of 10^3^–10^5^ Hz arose from the IBLC effect. This result is similar to that observed in previous studies [36]. Mostly, the ε′ decrease at high-frequencies was caused by the rotation of dipole moments that cannot be changed to follow the direction of the AC field; this is known as the dielectric relaxation behavior [6]. It is worth noting that after the sintering temperature increased, a step-like decrease in ε′ at a low-frequency range was not observed because the ε′ plateaued and the stepped decrease in ε′ shifted to a lower frequency. The shifting and plateauing of ε′ are consistent with the shift in the shoulder of the tanδ peak, which caused tanδ at 1 kHz to be lower than 0.05. The ε′ and tanδ values at 1 kHz for all ceramic samples are summarized in Table 2. The ε′ of WTO ceramics increased with increasing W^6+^ doping level concentration because the substitution of Ti^4+^ for W^6+^ generated free electrons in the TiO_2_ structure, as follows [29]:

(3) $${\mathrm{WO}}_{3}+{\mathrm{TiO}}_{2}\;\overset{{\mathrm{TiO}}_{2}}{\to }\;W_{\mathrm{Ti}}^{••}+2{\mathrm{Ti}}_{\mathrm{Ti}}^{\prime }+4O_{O}^{x}+\frac{1}{2}O_{2}↑$$

(4) $${\mathrm{Ti}}^{4+}+\;e\;¯\to {\;\mathrm{Ti}}^{3+}$$

According to Table 2, giant ε′ > 10^4^ values were obtained in all ceramic samples, whereas a low tanδ of <0.05 was achieved in only the 2.5%WTO-1500 ceramic. Usually, a high tanδ is found in the donor doped into TiO_2_ structures such as Nb^5+^ or Ta^5+^ single-doped TiO_2_ ceramics [8,23,31]. Moreover, tanδ decreased with increasing sintering temperature, which is similar to previous reports [31]. These results demonstrate that the ε′ and tanδ were controlled by the doping concentration and sintering temperature.

**Figure 6:**
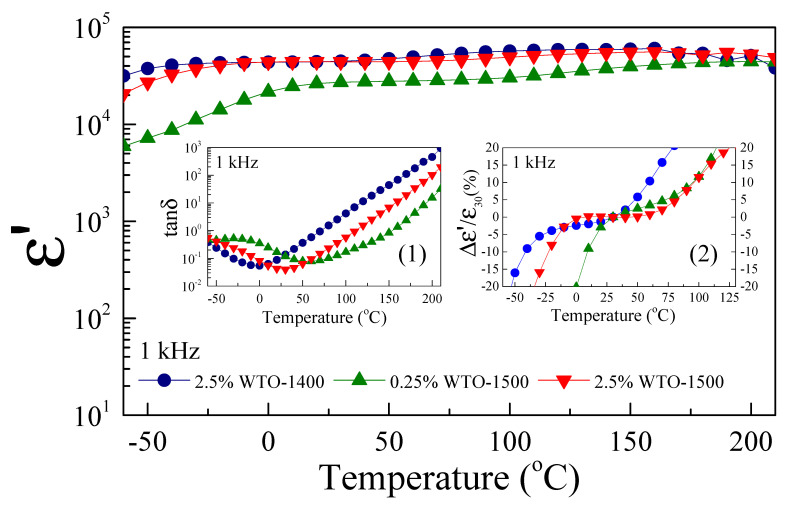
Temperature dependence of ε′ at 1 kHz of WTO ceramics; insets (1) and (2) show tanδ and temperature coefficient values.

Figure 6 displays the temperature dependence of the dielectric properties at 1 kHz. The ε′ drop at low temperatures correlated with the relaxation peak of tanδ (Inset (1)). The ε′ of the 0.25%WTO-1500 ceramic was strongly dependent on the temperature below 0 °C, while the ε′ of the 2.5%WTO-1500 ceramic slightly dropped below −25 °C. For temperature conditions, the ε′ of.2.5%WTO-1400 and 2.5%WTO-1500 dropped nearly in the same temperature range. The temperature coefficient of ε′ is shown in Inset (2). The results indicate that the temperature stability of ε′ can be improved by the doping conditions. As free electrons could be generated by W^6+^ doping into the TiO_2_ structure, the polarizability of WTO ceramics increased with increasing W^6+^ doping concentration. For this reason, the temperature stability ε′ of the 2.5%WTO-1500 ceramic at low temperatures is better than that of the 0.25%WTO-1500 ceramic.

**Figure 7:**
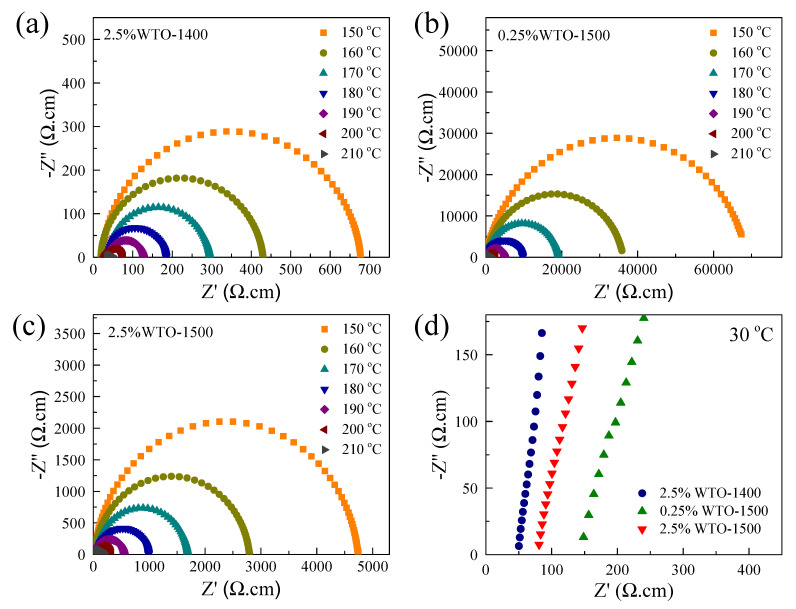
Impedance complex Z* plots with various temperatures of (**a**) 2.5%WTO-1400, (**b**) 0.25%WTO-1500, and (**c**) 2.5%WTO-1500 ceramics. (**d**) Nonzero intercept at the origin of WTO ceramics.

The origin of the giant dielectric properties of WTO ceramics was studied using impedance spectroscopy. As clearly seen in Figure 7a–c, a full semicircular arc was observed in the temperature range of 150–210 °C. Figure 7d displays the nonzero intercept on the *Z*-axis (at 30 °C) of WTO ceramics. For all ceramic samples, the diameter of the large semicircle arc decreased significantly with increasing temperature, which is similar to the results reported in the literature [21,31,38]. This observation indicated a decrease in resistance. Thus, this behavior correlates with an increase in tanδ in a high-temperature range. Based on impedance spectroscopy for polycrystalline ceramics [39,40], the diameter of a large semicircular arc and the nonzero intercept on the *Z*′ axis are related to the total resistance of the semiconducting part (R_s_) and insulating part (R_i_), respectively. It was observed that the R_i_ value of the 2.5%WTO-1500 ceramic was larger than that of the 2.5%WTO-1400 ceramic. This result indicates that the sintering temperature had a great effect on the formation of the insulating part in the WTO ceramics. At a high sintering temperature, more defects could be formed in the structure of the 2.5%WTO-1500 ceramic. Hence, charges could be confined to the local structure, giving rise to a decrease in conductivity. On the other hand, the R_i_ value of the 0.25%WTO-1500 ceramic was significantly larger than that of the 2.5%WTO-1500 ceramic. This result may be due to the different free charge concentrations due to the difference in doping concentration, following Equation (3). This result is related to the variation in the R_s_ values. The R_s_ value of the 0.25%WTO-1500 ceramic was the largest due to the lowest doping concentration. According to Maxwell–Wagner polarization [41], the frequency of the step-like decrease in the giant dielectric constant (f_c_) follows the relationship [36]:

(5) $$\tau \approx \;\frac{1}{2}{\pi f}_{c}\;\approx {\;R}_{s}C_{\mathrm{gb}}$$

where *τ* is the dielectric relaxation time. Thus, as demonstrated in Figure 5, the shifting of the step-like decrease in ε′ can be described by the change in the R_s_ value, which refers to the resistance of the grain (R_g_) in this work. Based on the dielectric property results, R_i_ may arise from the combined effects of the grain boundary and outer-surface layers. Therefore, the significant ε′ response of WTO ceramics can be described by the space charge polarization at the interface of semiconducting and insulating parts.

According to the impedance spectroscopy, the primary origin of the giant ε′ response was due to the interfacial polarization at the insulating layer, i.e., grain boundaries. Thus, the temperature stability is closely associated with the insulating properties of the insulating part based on the IBLC effect [21,22,23]. At high temperatures, under an applied electric field, the motion of free charges was inhibited at the insulating layers. Generally, the DC conduction is also the source of polarization in high temperatures, giving rise to the additional polarization and hence, increase in ε′. This is the primary cause of a high temperature coefficient at a high temperature. For the WTO ceramics, an improved temperature stability of the giant ε′ response at high temperatures was attributed to a large R_i_ value. Furthermore, free electrons that were confined to defect clusters in the TiO_2_ structure may also be a factor in the improved temperature stability [9].

## 4. Conclusions

A single-phase ceramic with a rutile-TiO_2_ structure was obtained for all the ceramic samples. The lattice parameters changed slightly with the W^6+^ doping level concentration but did not change with the sintering temperature. A dense microstructure without pores was observed. As the doping concentration and sintering temperature increased, the average grain size increased significantly. The dopant element was homogeneously dispersed in both the grains and grain boundaries. Oxygen vacancies were produced by sintering at a high temperature. Giant ε′ > 10^4^ was achieved, whereas a low tanδ of ~0.04 was only obtained in the 2.5%WTO-1500 ceramic. The WTO ceramics were electrically heterogeneous and consisted of semiconducting and insulating parts. The R_i_ value decreases significantly with increasing temperature. The R_s_ value is correlated with the frequency during a step-like decrease in ε′. Therefore, the large dielectric response of WTO ceramics can be described by the interfacial polarization between the semiconducting and insulating parts.

## Figures and Tables

**Table 1:** Lattice parameters (*a* and *c*) of WTO ceramics.

Samples	Lattice Parameters (Å)	
	*a*	*c*
TiO_2_	4.593(1)	2.962(2)
2.5%WTO-1400	4.592(9)	2.959(8)
0.25%WTO-1500	4.592(9)	2.960(6)
2.5%WTO-1500	4.593(0)	2.960(8)

**Table 2:** Dielectric properties (ε′ and tanδ) of all ceramic samples at room temperature (RT) and 1 kHz.

Samples	Dielectric Properties	
	ε′	tanδ
2.5%WTO-1200	33,066	0.565
2.5%WTO-1300	56,291	0.791
2.5%WTO-1400	44,830	0.138
0.25%WTO-1500	27,141	0.118
0.5%WTO-1500	40,438	0.227
2.5%WTO-1500	44,208	0.040

## Data Availability

The data presented in this study are available in article.
